# Rosemary Flowers as Edible Plant Foods: Phenolic Composition and Antioxidant Properties in *Caenorhabditis elegans*

**DOI:** 10.3390/antiox9090811

**Published:** 2020-09-01

**Authors:** Cristina Moliner, Víctor López, Lillian Barros, Maria Inês Dias, Isabel C. F. R. Ferreira, Elisa Langa, Carlota Gómez-Rincón

**Affiliations:** 1 Department of Pharmacy, Faculty of Health Sciences, Universidad San Jorge, 50830 Villanueva de Gállego (Zaragoza), Spain; acmoliner@usj.es (C.M.); ilopez@usj.es (V.L.); elanga@usj.es (E.L.); 2 Instituto Agroalimentario de Aragón-IA2 (CITA-Universidad de Zaragoza), 50013 Zaragoza, Spain; 3 Centro de Investigação de Montanha (CIMO), Instituto Politécnico de Bragança, Campus de Santa Apolónia, 5300-253 Bragança, Portugal; lillian@ipb.pt (L.B.); maria.ines@ipb.pt (M.I.D.)

**Keywords:** rosemary, laminaceae, edible flowers, polyphenols, antioxidant activity, functional foods

## Abstract

*Rosmarinus officinalis* L., commonly known as rosemary, has been largely studied for its wide use as food ingredient and medicinal plant; less attention has been given to its edible flowers, being necessary to evaluate their potential as functional foods or nutraceuticals. To achieve that, the phenolic profile of the ethanolic extract of *R. officinalis* flowers was determined using LC-DAD-ESI/MSn and then its antioxidant and anti-ageing potential was studied through in vitro and in vivo assays using *Caenorhabditis elegans*. The phenolic content was 14.3 ± 0.1 mg/g extract, *trans* rosmarinic acid being the predominant compound in the extract, which also exhibited a strong antioxidant capacity in vitro and increased the survival rate of *C. elegans* exposed to lethal oxidative stress. Moreover, *R. officinalis* flowers extended *C. elegans* lifespan up to 18%. Therefore, these findings support the potential use of *R. officinalis* flowers as ingredients to develop products with pharmaceutical and/or nutraceutical potential.

## 1. Introduction

For many years, flowers have been used for a large number of applications including ornamental, medicinal, cosmetic and culinary purposes. In Europe, edible flowers, such as roses, calendula or saffron, have been consumed from ancient times [1]. Literature about these edible species is scarce, however they are gaining attention as functional foods and as nutraceuticals [2].

In general, the nutritional composition of flowers is very similar to other plant organs [1]; by contrast, phytochemical constituents of flowers can differ from them [3,4]. These phytochemicals are mainly phenolic compounds and are responsible for the health benefits associated with flowers such as antioxidant, anti-inflammatory or neuroprotective effects [5]. Thus, the assessment of their chemical composition and bioactive characteristics needs to be studied, particularly the impact of these edible flowers on human health.

*Rosmarinus officinalis* L. (rosemary) is a traditional plant native to the Mediterranean area belonging to the *Lamiaceae* family. This species is widely used in gastronomy, commonly consumed as a spice or culinary herb, and may be less known as an edible flower [6,7]. Most of the previous research is focused on its leaves and its essential oil activity, which have been used as therapeutic agents since ancient times [8], while the composition and bioactive potential of its flowers remain understudied.

The nematode *Caenorhabditis elegans* makes it possible to study the effects of natural products in an in vivo model, especially for testing their antioxidant capacities and their influence on longevity. This is due, in part, to the high similarity of its genome to the human one, including stress response pathways. Nearly 60–80% of human genes have homologues in *C. elegans* [9]. Moreover, this model is also very popular, among other reasons, because it has a short lifespan and can be easily maintained in the laboratory [10].

Considering all the above, the aim of this study was to evaluate, for the first time, the antioxidant and anti-ageing potential of rosemary flower ethanolic extract using in vivo tests on *Caenorhabditis elegans*, and also to determine its phytochemical composition regarding phenolic compounds through LC-DAD-ESI/MSn.

## 2. Materials and Methods

### 2.1. Standards and Reagents

DPPH (2,2-diphenyl-1-picrylhydrazyl), formic acid, and pyrogallol were purchased from Sigma-Aldrich (St. Louis, MO, USA). Juglone (5-hydroxy-1,4-naphthoquinone) was from Alfa Aesar (Ward Hill, MA, USA), Folin–Ciocalteu reagent was purchased from Chem-lab (Zeldelgem, Belgium). Phenolic compounds (apigenin-7-*O*-glucoside, chlorogenic acid, p-coumaric acid, caffeic acid, isorhamnetin-3-*O*-rutinoside, isorhamnetin-3-*O*-glucoside, quercetin-3-*O*-glucoside, and rosmarinic acid) were from Extrasynthese (Genay, France). Water was treated in a Milli-Q water purification system (TGI Pure Water Systems, Greenville, SC, USA). Other solvents and reagents were acquired from common sources.

### 2.2. Samples and Preparation of Extract

*Rosmarinus officinalis* L. flowers used in this study were collected in March 2016 in Herrera de los Navarros (Zaragoza, Spain). Voucher specimens were deposited in the herbarium of San Jorge University. Ethanolic extract of fresh flowers was obtained by percolation with a Soxhlet apparatus for 4 h. The extract was concentrated to dryness with a rotary flash evaporator Buchi and stored at −20 °C for further study.

### 2.3. Analysis of Phenolic Compounds

A Dionex Ultimate 3000 UPLC (Thermo Scientific, San Jose, CA, USA) chromatographic system was used to profile the phenolic composition of *R. officinalis* flowers’ ethanolic extract. These compounds were separated and identified as previously described by Bessada et al. [11], after re-dissolving the extract at a concentration of 10 mg/mL with an ethanol:water (80:20, *v*/*v*) mixture. A double online detection was performed using a DAD (280, 330 and 370 nm as preferred wavelengths) and a mass spectrometer equipped with an ESI source (MS detection performed in negative mode, Linear Ion Trap LTQ XL, Thermo Finnigan, San Jose, CA, USA).

Phenolic compounds identification was performed based on their chromatographic behavior and UV-Vis and mass spectra by comparison with standard compounds, when available, and data reported in the literature giving a tentative identification. Data acquisition was carried out with the Xcalibur^®^ data system (Thermo Finnigan, San Jose, CA, USA). A calibration curve for each available phenolic standard was constructed based on the UV-Vis signal, for the quantification analysis. A manual quantification was performed using the baseline to valley integration with baseline projection mode in order to calculate peak areas. For the identified phenolic compounds for which a commercial standard was not available, the most similar available standard was applied. The results were expressed as mg/g of extract.

### 2.4. C. elegans Assays

#### 2.4.1. *C. elegans* Strains and Maintenance

The strains used in this study were obtained from the *Caenorhabditis* Genetics Center, CGC (Minneapolis, MN, USA). N2 (wild type) worms were grown and maintained at 20 °C, while strain SS104 *glp-4(bn2*) was maintained at 16 °C. Both *C. elegans* strains were routinely propagated on Nematode Growth Medium (NGM) (bioWORLD, Ohio, OH, USA) plates with a lawn of *Escherichia coli* OP50 (CGC, Minneapolis, MN, USA). Unless stated otherwise, age-synchronized populations were obtained by sodium hypochlorite treatment of gravid adults according to standardized methods [12].

#### 2.4.2. Acute Toxicity Assay

The Donkin and Williams method was followed with some modifications [13]. Synchronized L4 were washed twice with M9 buffer and re-suspended in K-medium (32 mM KCl, 51 mM NaCl). A quantity of 200 μL of this suspension was transferred to each well of the 96-well plates (7–18 worms/well) and mixed with 50 μL of diluted extract or K-medium as negative control. After 24 h at 20 °C, survival was measured. The results were expressed as percentage of survival rate (% Survival rate) or viability (Equation (1)):

% Survival rate = (Number of alive worms × 100)/Total number of worms(1)

At least 40 worms per condition were evaluated in each assay.

#### 2.4.3. Oxidative Stress Resistance Assay

Oxidative stress resistance assay was based on the method described by Surco-Laos et al. with modifications [14]. Synchronized L1 worms were cultured for 48 h at 20 °C in NGM plates containing different concentrations of flower extract (50–500 μg/mL). Then, worms were washed twice with sterile water and transferred to a microtiter plate containing NGM agar with 150 μM juglone, which induced lethal oxidative stress. After 24 h of exposition, survival was scored. A worm was considered to be dead when it did not respond to gentle touch with a platinum wire. Results were represented as a % Survival rate and calculated using Equation (1). For each assay, about 120 individuals were used per studied condition.

#### 2.4.4. Lifespan Assay

Lifespan assay was carried out according to Virk et al. on *C. elegans* SS104 *glp-4 (bn2)* [15], which is a temperature-sensitive strain that does not produce progeny at restrictive temperature (25 °C). Egg lay were performed to obtain synchronized populations. Eggs were raised at 16 °C until the L4 stage; at that time, worms were moved into a 25 °C environment. After 24 h, 25 worms were placed onto NGM fresh plates containing the extract (25–250 μg/mL) or in absence of it (control group). A total of five plates per condition were used. This moment was considered the day 0 for the counting of surviving worms. Worms were moved to new plates after 7 and 14 days while scoring for survival every two or three days. The scoring method was the same described in Section 2.4.3.

### 2.5. In Vitro Reducing/Antiradical Activity

The antioxidant capacity of the extract of rosemary flowers was determined by the Folin–Ciocalteu reagent assay (Total Phenolic Content, TPC) [16], the DPPH· radical scavenging activity assay [17] and the ferric reducing antioxidant power (FRAP) assay [18]. The results were expressed as mg of pyrogallol equivalent per g of extract (mg PE/g extract), radical scavenging activity (%) and μmol Fe^2+^/g extract, respectively.

### 2.6. Statistical Analyses

Three replicates were carried out for all the performed assays. Results were represented as mean ± SEM. Statistical analyses of the data were performed with GraphPad Prism version 6.0c for Mac (GraphPad Software, San Diego, CA, USA). The 50% inhibitory concentration (IC_50_) was estimated by a nonlinear regression for DPPH^·^ assays. The results of toxicity and resistance to oxidative stress assays were analyzed using ANOVA followed by Tukey’s multiple comparisons test. For lifespan assay, the Kaplan–Meier survival model was utilized, and *p* values were calculated using the log-rank test. *p* ≤ 0.05 was considered statistically significant.

## 3. Results and Discussion

### 3.1. Phenolic Compounds of Rosemary Flowers

**Figure 1:**
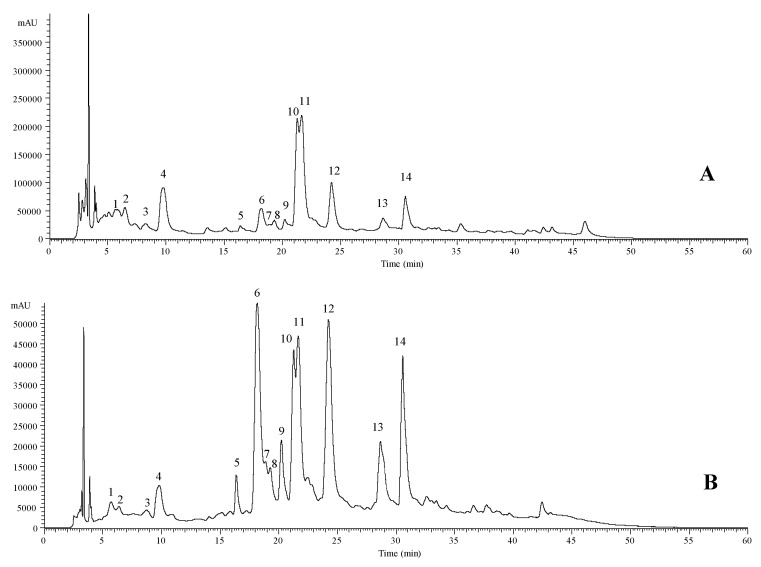
Phenolic profile of *R. officinalis* flowers recorded at 280 nm (**A**) and 370 nm (**B**). Numbers 1 to 14 refer to peaks from Table 1.

The obtained extract from fresh flowers of *R. officinalis* had a yield of 3.75% (mass of extract/mass of fresh flowers) and the characterization of the phenolic compounds present in it was performed by LC-DAD-ESI/MSn. The analysis of the extract revealed the presence of 14 compounds, corresponding to six phenolic acids and eight flavonoids (Table 1). The representative chromatogram is shown in Figure 1.

4-*O*-caffeoylquinic acid (2 isomers), medioresinol (phenolic lignin), caffeic acid and rosmarinic acid (2 isomers) were the identified phenolic acids, whereas the flavonoid compounds found were glycoside derivates of luteolin (5 compounds), isorhamnetin (2 compounds) and quercetin (1 compound). All the mentioned compounds, with the exception of the phenolic lignin and isorhamnetin glycoside derivatives, have been previously described by the authors in other studies involving *R. officinalis* [19,20,21]. Thus, peak 3 ([M − H]^−^ ion at *m*/*z* 387) presented two main MS^2^ fragment ions at *m/z* 207 and 163, being tentatively identified as medioresinol, taking into account data reported in the literature [22,23,24]. This compound has been previously identified in the hydroethanol and acetone-based extracts of *R. officinalis* leaves [22,24] and in the methanolic extract of *Bituminaria bituminos* (L.) C.H. Stirton flowers [23]. Peaks 8 ([M − H]^−^ ion at *m/z* 623) and 9 ([M − H^]−^ ion at *m/z* 477) were positively identified as isorhamnetin-3-*O*-rutinoside and isorhamnetin-3-*O*-glucoside in comparison with the commercial standard. Nevertheless, most of the identified compounds have been previously identified by other authors in different plant parts of *R. officinalis* [22,24,25,26,27,28].

The three major components, ordered from the highest to the lowest concentration, were identified as *trans* rosmarinic acid (peak 11), *cis* rosmarinic acid (peak 10) and luteolin-*O*-glucuronide (peak 12), respectively (Table 1). As reported by Del Baño et al., rosmarinic acid was the major compound found in the flowers [3] and was at a slightly higher content than the one described by Moreno, Scheyer, Romano, and Vojnov [29]. However, most of the compounds detected in the extract, such as caffeic acid, medioresinol, luteolin, isorhamnetin and quercetin glycoside derivatives, have been previously identified in different organs of *R. officinalis* [3,4,6,21,30], but not in its flowers.

On the other hand, the polyphenol content of the extract was higher than that described by Chen et al. for a sample obtained from dried flowers using methanol–acetone–water as solvent and ultrasound extraction [31]. These differences could be due to different geographical origins of the plant material, or different geo-climatic conditions, and also different harvest times, but they could also be explained by the application of different solvents and extraction systems. Thus, this result demonstrated that *R. officinalis* flowers are a noticeable source of phenolic compounds.

### 3.2. Evaluation of Rosemary Flowers Acute Toxicity

Previous studies have demonstrated the lack of toxicity of extracts obtained from leaves of *R. officinalis*, which are used as a food additive [32]; however, there is no data available about the toxicity of its flowers.

Worms exposed to a concentration range of 10–2000 μg/mL for 24 h did not significantly reduce viability, even at the highest tested dose. *C. elegans* maintained a viability rate of 96% ± 2 while the viability of the control group was 96% ± 3. Thus, *R. officinalis* flowers at the assayed concentrations did not produce lethal toxicity on this model organism. It should be noted that *C. elegans* is a powerful tool in the toxicology field. Screenings using this organism have shown to be a predictive tool when measuring rat or mouse LD_50_ [33].

### 3.3. Evaluation of Protective Effects on C. elegans under Lethal Oxidative Stress

**Figure 2:**
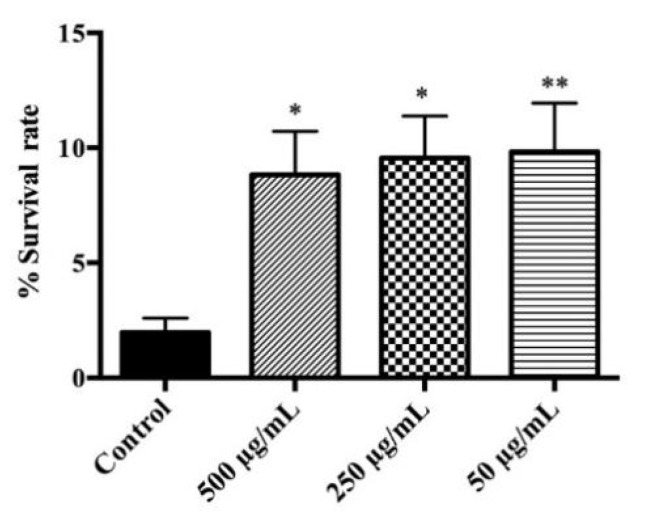
Effect of *Rosmarinus officinalis* flower extract on the response to a lethal oxidative stress induced by juglone (150 µM) on *C. elegans.* Control (*n* = 383), 500 (*n* = 399), 250 (*n* = 327) and 50 (*n* = 391) µg/mL groups, where *n* = number of individuals assayed. Differences compared to the control group were considered significant at *p* ≤ 0.05 (*) and *p* ≤ 0.01 (**).

We analyzed the effect of the studied extract on the resistance of *C. elegans* against lethal oxidative stress induced by juglone. This natural compound is a strong pro-oxidant, which generates Reactive Oxygen Species (ROS), inducing cell death [34]. As shown in Figure 2, the pre-treatment with *R. officinalis* flower extract increased survival of *C. elegans* compared to the control group. The best protective effect was found in groups pre-treated with 50 (*p* ≤ 0.01) and 250 (*p* ≤ 0.05) μg/mL of extract, for which the survival rate was increased from 2.0% ± 0.6 (control group) to 10% ± 2. These results are in concordance with the study carried out by Zamberlan et al., which evaluated the effect of an extract obtained from dried leaves of *R. officinalis* on stress resistance induced with juglone [35]. The enhanced response to oxidative stress of *R. officinalis* could be attributed to the presence of phenolic acids and flavonoids in the extracts, which are considered to be the main compounds responsible for the antioxidant activity of *R. officinalis*, such as rosmarinic acid [36]. Lin et al. reported an increased survival rate in nematodes pre-treated with this phenolic acid and exposed to paraquat-induced oxidative stress compared to the control group [37].

### 3.4. Evaluation of C. elegans Lifespan

**Figure 3:**
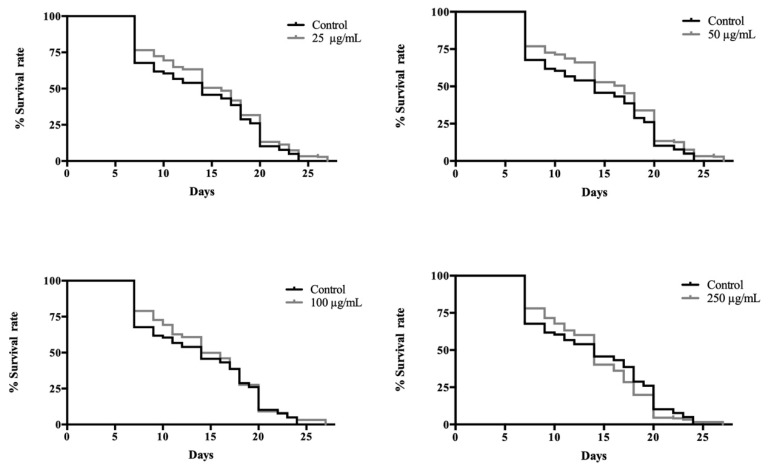
Effect of *Rosmarinus officinalis* flower extract on lifespan of *C. elegans* SS104 *glp-4 (bn2)*. The means of lifespan were: 14 days (control, 100 and 250 µg/mL groups), 16 days (25 µg/mL treated group) and 17 days (50 µg/mL treated group). Results of lifespan experiments were analysed using the Kaplan–Meier survival model, and for significance by means of a long rank pairwise comparison test between the control and treatment groups. Differences in survival curves between the treatment and control groups were found for the concentrations of 25 (*p* ≤ 0.05) and 50 (*p* ≤ 0.01) µg/mL.

In order to determine the impact of the flowers’ extract on the life extension, a lifespan assay was performed at 25 °C using SS104 *glp-4 (bn2)* worms, which were exposed to different concentrations of *R. officinalis* flowers’ ethanolic extract. As shown in Figure 3, *R. officinalis* flowers had a noteworthy effect on lifetime at concentrations of 25 (*p* ≤ 0.05) and 50 (*p* ≤ 0.01) µg/mL. The best result was found in the group treated with 50 µg/mL, showing an increase of around 18% of median lifespan regarding the untreated group.

The present study does not allow us to conclude which mechanisms are involved in lifespan lengthening, but similar experiments were carried out with an extract of *R. officinalis* leaves and with the major phytochemicals present in this extract, as described later. The life-extending effect could be due to up-regulating stress-resistance-associated genes such as DAF-16 and HSP-1, associated with the insulin/IGF (insulin-like growth factor) signaling (IIS) pathway [35,37,38,39]. The IIS pathway is a key regulator of aging and longevity in many organisms, including *C. elegans* [40].

### 3.5. In Vitro Reducing/Antioxidant Activity

In order to complete the antioxidant potential of fresh flowers of *R. officinalis*, different experiments were carried out: Folin–Ciocalteu (TPC), DPPH radical scavenging, and FRAP assays. Results are shown in Table 2.

Firstly, TPC was determined by the Folin–Ciocalteu method, which is based on a redox reaction, and the result was expressed in comparison to pyrogallol standard. Our extract contained 48 ± 2 mg PE/g extract. This value is considerably higher than the one quantified by LC-DAD-ESI/MSn, which can be attributed to the capacity of non-phenolic compounds (e.g., proteins, thiols or vitamins) to react with the Folin–Ciocalteu reagent [41]. Therefore, TPC assay is considered as a measure of an overall antioxidant capacity.

The IC_50_ value of *R. officinalis* flower extract for scavenging DPPH radicals was 67 ± 5 µg/mL (Table 2). The reducing power of the sample, as measured by FRAP assay, was 34 ± 2 µmol Fe^2+/^g extract. Our findings revealed that *R. officinalis* fresh flowers exhibited important phenolic content with strong scavenging power when compared with the values reported for other flower species [42].

Several studies have demonstrated the beneficial impact of dietary intake of phenolic compounds in health to a large extent due to their antioxidant properties. Polyphenols protect against diseases associated with aging such as cardiovascular diseases, inflammation or neurodegeneration [43]. The ageing of the population has increased the incidence of these problems and, as a consequence, it is necessary to design new strategies to counteract them. Therefore, the assessment of bioactivities of edible flowers, which are rich in phenolic compounds, can contribute to achieve this goal.

## 4. Conclusions

To summarize, our study reveals that *R. officinalis* edible flowers are a good source of phenolic compounds, in which rosmarinic acid was identified as the main phenolic compound. In addition, for the first time it has been found that *R. officinalis* flowers possess both anti-aging and anti-oxidative activities in vivo on *C. elegans*. Hence, this study promotes further research into the precise physiological and molecular signaling mechanisms of *R. officinalis* flowers on elongated lifespan and increased oxidative stress resistance in *C. elegans*.

## Figures and Tables

**Table 1:** Retention time (Rt), wavelengths of maximum absorption in the visible region (λ_max_), mass spectral data, tentative identification and quantification (mg/g of extract) of the phenolic compounds present in *Rosmarinus officinalis* L. flowers.

Peak	Rt (min)	λ_max_ (nm)	Molecular Ion [M − H]^−^ (*m/z*)	MS^2^ (*m/z*)	Tentative Identification	Quantification (mg/g of Extract)
**1**	5.65	322	353	191(20),179(57),173(100),155(5),135(10)	*cis* 4-*O*-Caffeoylquinic acid ^A^	0.656 ± 0.003
**2**	6.47	324	353	191(17),179(52),173(100),155(3),135(8)	*trans* 4-*O*-Caffeoylquinic acid ^A^	0.91 ± 0.03
**3**	8.27	312	387	207(100),179(5),163(42)	Medioresinol ^B^	0.28 ± 0.02
**4**	9.8	327	179	135(100)	Caffeic acid ^C^	0.76 ± 0.02
**5**	16.39	340	609	285(100)	Luteolin-*O*-di-hexoside ^D^	0.52 ± 0.01
**6**	18.17	245/266/345	461	285(100)	Luteolin-7-*O*-glucuronide ^D^	0.99 ± 0.01
**7**	18.96	350	463	301(100)	Quercetin-3-*O*-glucoside ^E^	0.54 ± 0.01
**8**	19.28	350	623	315(100),301(42)	Isorhamnetin-3-*O*-rutinoside ^E^	0.55 ± 0.01
**9**	20.26	350	477	315(100)	Isorhamnetin-3-*O*-glucoside ^E^	0.62 ± 0.02
**10**	21.25	327	359	197(36),179(42),161(100),135(5)	*cis* Rosmarinic acid ^F^	2.64 ± 0.02
**11**	21.68	328	359	197(33),179(44),161(100),135(5)	*trans* Rosmarinic acid ^F^	3.4 ± 0.1
**12**	24.25	345	461	285(100)	Luteolin-*O*-glucuronide ^D^	1.03 ± 0.01
**13**	28.68	332	503	285(100)	Luteolin-3′-acetyl-*O*-glucuronide ^D^	0.60 ± 0.03
**14**	30.57	330	503	285(100)	Luteolin-3′-acetyl-*O*-glucuronide ^D^	0.9 ± 0.1
**Total phenolic acids**						**8.69 ± 0.05**
**Total flavonoids**						**5.69 ± 0.04**
**Total phenolic compounds**						**14.3 ± 0.1**

Letters correspond to the standard calibration curves: A—chlorogenic acid (*y* = 168823x − 161172, *R*^2^ = 0.9999; LOD = 0.20 µg/mL; LOQ = 0.68 µg/mL), B—*p*-coumaric acid (*y* = 301950x + 6966.7, *R*^2^ = 0.999; LOD = 0.68 μg/mL; LOQ = 1.61 μg/mL), C—caffeic acid (*y* = 388345x + 406369; *R*^2^ = 0.994; LOD = 0.19 µg/mL; LOQ = 0.65 µg/mL), D—apigenina-7-*O*-glucoside (y = 10683x − 45794; *R*^2^ = 0.999; LOD = 0.10 μg/mL; LOQ = 0.53 μg/mL); E—quercetin 3-*O*-glucoside (*y* = 34843x − 160173, *R*^2^ = 0.999; LOD = 0.21 µg/mL; LOQ = 0.71 µg/mL); F—rosmarinic acid (*y* = 191291x − 652903, *R*^2^ = 0.999; LOD = 0.15 µg/mL; LOQ = 0.68 µg/mL).

**Table 2:** Reducing/antiradical activity of *Rosmarinus officinalis* L. flowers’ extract.

Assay	Mean ± SEM
**TPC** mg PE/g extract	48 ± 2
**DPPH** IC_50_ [µg/mL]	67 ± 5
**FRAP** µmol Fe^2+^/g extract	34 ± 2

TPC, Total Phenolic Content; DPPH, 2,2-diphenyl-1-picrylhydrazyl; FRAP, ferric reducing antioxidant power.

## References

[1] Mlcek J., Rop O. (2011). Fresh edible flowers of ornamental plants—A new source of nutraceutical foods. Trends Food Sci. Technol..

[2] Takahashi J.A., Rezende F.A.G.G., Moura M.A.F., Dominguete L.C.B., Sande D. (2020). Edible flowers: Bioactive profile and its potential to be used in food development. Food Res. Int..

[3] Del Baño M.J., Lorente J., Castillo J., Benavente-García O., Del Río J.A., Ortuño A., Quirin K.-W., Gerard D. (2003). Phenolic diterpenes, flavones, and rosmarinic acid distribution during the development of leaves, flowers, stems, and roots of *Rosmarinus officinalis*. Antioxidant activity. J. Agric. Food Chem..

[4] Del Baño M.J., Lorente J., Castillo J., Benavente-García O., Marín M.P., Del Río J.A., Ortuño A., Ibarra I. (2003). Flavonoid Distribution during the Development of Leaves, Flowers, Stems, and Roots of *Rosmarinus officinalis*. Postulation of a Biosynthetic Pathway. J. Agric. Food Chem..

[5] Lu B., Li M., Yin R. (2016). Phytochemical Content, Health Benefits, and Toxicology of Common Edible Flowers: A Review. Crit. Rev. Food Sci. Nutr..

[6] Loizzo M.R., Pugliese A., Bonesi M., Tenuta C., Menichini F., Xiao J., Tundis R. (2015). Edible Flowers: A Rich Source of Phytochemicals with Antioxidant and Hypoglycaemic Activity. J. Agric. Food Chem..

[7] Wang F., Miao M., Xia H., Yang L.-G., Wang S.-K., Sun G.J. (2017). Antioxidant activities of aqueous extracts from 12 Chinese edible flowers in vitro and in vivo. Food Nutr. Res..

[8] Committee on Herbal Medicinal Products (HMPC) (2010). Assessment Report on Rosmarinus officinalis L., Aetheroleum and Rosmarinus officinalis L., Folium.

[9] Calvo D.R., Martorell P., Genovés S., Gosálbez L. (2016). Development of novel functional ingredients: Need for testing systems and solutions with Caenorhabditis elegans. Trends Food Sci. Technol..

[10] Martorell P., Llopis S., Gonza N., Monto F., Ortiz P., Genove S. (2012). Caenorhabditis elegans as a Model to Study the E FF ectiveness and Metabolic Targets of Dietary Supplements Used for Obesity Treatment: The Speci FI C Case of a Conjugated Linoleic Acid Mixture (Tonalin). J. Agric. Food Chem..

[11] Bessada S., Barreira J.C., Barros L., Ferreira I.C., Oliveira M.B.P. (2016). Phenolic profile and antioxidant activity of *Coleostephus myconis* (L.) Rchb. F.: An underexploited and highly disseminated species. Ind. Crop. Prod..

[12] Stiernagle T. (1999). Maintenance of C. elegans. WormBook.

[13] Donkin S.G., Williams P.L. (1995). Influence of developmental stage, salts and food presence on various end points using *Caenorhabditis Elegans* for aquatic toxicity testing. Environ. Toxicol. Chem..

[14] Surco-Laos F., Cabello J., Gómez-Orte E., González-Manzano S., González-Paramás A.M., Santos-Buelga C., Dueñas M., Dueñas M. (2011). Effects of O-methylated metabolites of quercetin on oxidative stress, thermotolerance, lifespan and bioavailability on *Caenorhabditis elegans*. Food Funct..

[15] Virk B., Correia G.D.S., Dixon D., Feyst I., Jia J., Oberleitner N., Briggs Z., Hodge E., Edwards R., Ward J.M. (2012). Excessive folate synthesis limits lifespan in the *C. elegans*: *E. coli* aging model. BMC Biol..

[16] Singleton V.L., Rossi J.A. (1965). Colorimetry of total phenolics with phosphomolybdic-phosphotungstic acid reagents. Am. J. Enol. Vitic..

[17] López V., Akerreta S., Casanova E., García-Mina J., Cavero R., Calvo M. (2008). Screening of Spanish Medicinal Plants for Antioxidant and Antifungal Activities Screening of Spanish Medicinal Plants for Antioxidant and Antifungal Activities. Pharm. Biol..

[18] Pulido R., Bravo L., Saura-Calixto F. (2000). Antioxidant Activity of Dietary Polyphenols As Determined by a Modified Ferric Reducing / Antioxidant Power Assay. J. Agric. Food Chem..

[19] Ribeiro A., Caleja C., Barros L., Santos-Buelga C., Barreiro M.F., Ferreira I.C. (2016). Rosemary extracts in functional foods: Extraction, chemical characterization and incorporation of free and microencapsulated forms in cottage cheese. Food Funct..

[20] Gonçalves G.D.A., De Sá-Nakanishi A.B., Comar J.F., Bracht L., Dias M.I., Barros L., Peralta R.M., Ferreira I.C., Bracht A. (2018). Water soluble compounds of: *Rosmarinus officinalis* L. improve the oxidative and inflammatory states of rats with adjuvant-induced arthritis. Food Funct..

[21] Gonçalves G.A., Corrêa R.C., Barros L., Dias M.I., Calhelha R.C., Correa V.G., Bracht A., Peralta R.M., Ferreira I.C. (2019). Effects of in vitro gastrointestinal digestion and colonic fermentation on a rosemary (*Rosmarinus officinalis* L.) extract rich in rosmarinic acid. Food Chem..

[22] Ożarowski M., Mikołajczak P.Ł., Bogacz A., Gryszczyńska A., Kujawska M., Jodynis-Liebert J., Piasecka A., Napieczynska H., Szulc M., Kujawski R. (2013). *Rosmarinus officinalis* L. leaf extract improves memory impairment and affects acetylcholinesterase and butyrylcholinesterase activities in rat brain. Fitoterapia.

[23] Llorent-Martínez E.J., Spínola V., Gouveia S., Castilho P. (2015). HPLC-ESI-MSn characterization of phenolic compounds, terpenoid saponins, and other minor compounds in *Bituminaria bituminosa*. Ind. Crop. Prod..

[24] Mena P., Cirlini M., Tassotti M., Herrlinger K.A., Dall’Asta C., Del Rio D. (2016). Phytochemical profiling of flavonoids, phenolic acids, terpenoids, and volatile fraction of a rosemary (*Rosmarinus officinalis* L.) extract. Molecules.

[25] Linares I.B., Arráez-Román D., Herrero M., Ibanez E., Segura-Carretero A., Fernández-Gutiérrez A. (2011). Comparison of different extraction procedures for the comprehensive characterization of bioactive phenolic compounds in *Rosmarinus officinalis* by reversed-phase high-performance liquid chromatography with diode array detection coupled to electrospray time. J. Chromatogr. A.

[26] Mulinacci N., Innocenti M., Bellumori M., Giaccherini C., Martini V., Michelozzi M. (2011). Storage method, drying processes and extraction procedures strongly affect the phenolic fraction of rosemary leaves: An HPLC/DAD/MS study. Talanta.

[27] Borrás-Linares I., Stojanovic Z., Quirantes-Piné R., Arráez-Román D., Švarc-Gajić J., Fernández-Gutiérrez A., Segura-Carretero A. (2014). *Rosmarinus officinalis* leaves as a natural source of bioactive compounds. Int. J. Mol. Sci..

[28] Ferrer-Gallego P.P., Ferrer-Gallego R., Roselló R., Peris J.B., Guillén A., Gómez J., Laguna E. (2014). A new subspecies of *Rosmarinus officinalis* (Lamiaceae) from the eastern sector of the Iberian Peninsula. Phytotaxa.

[29] Moreno S., Scheyer T., Romano C.S., Vojnov A.A. (2006). Antioxidant and antimicrobial activities of rosemary extracts linked to their polyphenol composition. Free. Radic. Res..

[30] Ribeiro-Santos R., Carvalho-Costa D., Cavaleiro C., Costa H.S., Albuquerque T.G., Castilho M.C., Ramos F., Melo N.R., Sanches-Silva A. (2015). A novel insight on an ancient aromatic plant: The rosemary (*Rosmarinus officinalis* L.). Trends Food Sci. Technol..

[31] Chen G.-L., Chen S.-G., Xiao Y., Fu N.-L. (2018). Antioxidant capacities and total phenolic contents of 30 flowers. Ind. Crop. Prod..

[32] EFSA Panel on Food Additives and Nutrient Sources added to Food (EFSA ANS Panel) (2018). Refined exposure assessment of extracts of rosemary (E 392) from its use as food additive. EFSA J..

[33] Hunt P.R. (2017). The *C*. elegans model in toxicity testing. J. Appl. Toxicol..

[34] Pluci B., Kuczy P. (2017). The oxidative stress in allelopathy: Participation of prenyllipid antioxidants in the response to juglone in Chlamydomonas reinhardtii. Phytochemistry.

[35] Zamberlan D., Amaral G., Arantes L.P., Machado M.L., Mizdal C., De Campos M.M.A., Soares F.A.A. (2016). *Rosmarinus officinalis* L. increases *Caenorhabditis elegans* stress resistance and longevity in a DAF-16, HSF-1 and SKN-1-dependent manner. Braz. J. Med. Biol. Res..

[36] Frankel E.N., Huang S.-W., Aeschbach R., Prior E. (1996). Antioxidant Activity of a Rosemary Extract and Its Constituents, Carnosic Acid, Carnosol, and Rosmarinic Acid, in Bulk Oil and Oil-in-Water Emulsion. J. Agric. Food Chem..

[37] Lin C., Xiao J., Xi Y., Zhang X., Zhong Q., Zheng H., Cao Y., Chen Y.-J. (2019). Rosmarinic acid improved antioxidant properties and healthspan via the IIS and MAPK pathways in *Caenorhabditis elegans*. BioFactors.

[38] Wang F., Liu Q.D., Wang L., Zhang Q., Hua Z.T. (2011). The Molecular Mechanism of Rosmarinic Acid Extending the Lifespan of *Caenorhabditis elegans*. Appl. Mech. Mater..

[39] Pietsch K., Saul N., Chakrabarti S., Sturzenbaum S., Menzel R., Steinberg C.E.W. (2011). Hormetins, antioxidants and prooxidants: Defining quercetin-, caffeic acid- and rosmarinic acid-mediated life extension in *C. elegans*. Biogerontology.

[40] Jęsko H., Stępień A., Lukiw W.J., Strosznajder R.P. (2019). The Cross-Talk Between Sphingolipids and Insulin-Like Growth Factor Signaling: Significance for Aging and Neurodegeneration. Mol. Neurobiol..

[41] Everette J.D., Bryant Q.M., Green A.M., Abbey Y.A., Wangila G.W., Walker R.B. (2014). A thorough study of reactivity of various compound classes towards the Folin-Ciocalteu. J. Agric. Food Chem..

[42] Li A.-N., Li S., Li H.-B., Xu N.-P., Xu X.-R., Chen F. (2014). Total phenolic contents and antioxidant capacities of 51 edible and wild flowers. J. Funct. Foods..

[43] Del Bo’ C., Bernardi S., Marino M., Porrini M., Tucci M., Guglielmetti S., Cherubini A., Carrieri B., Kirkup B.M., Kroon P. (2019). Systematic Review on Polyphenol Intake and Health Outcomes: Is there Sufficient Evidence to Define a Health-Promoting Polyphenol-Rich Dietary Pattern?. Nutrients.

